# Rodent models of impaired fear extinction

**DOI:** 10.1007/s00213-018-5054-x

**Published:** 2018-10-31

**Authors:** Nicolas Singewald, Andrew Holmes

**Affiliations:** 1 Department of Pharmacology and Toxicology, Institute of Pharmacy, Center for Molecular Biosciences Innsbruck, University of Innsbruck, Innsbruck, Austria; 2 Laboratory of Behavioral and Genomic Neuroscience, National Institute on Alcohol Abuse and Alcoholism, NIH, Bethesda, MD USA

**Keywords:** Threat conditioning, Deficient fear inhibition, Anxiety drug development, Sex differences, Fear extinction, Cognitive behavioral therapy, Medial prefrontal cortex, Amygdala

## Abstract

The measurement of Pavlovian forms of fear extinction offers a relatively simple behavioral preparation that is nonetheless tractable, from a translational perspective, as an approach to study mechanisms of exposure therapy and biological underpinnings of anxiety and trauma-related disorders such as post-traumatic stress disorder (PTSD). Deficient fear extinction is considered a robust clinical endophenotype for these disorders and, as such, has particular significance in the current “age of RDoC (research domain criteria).” Various rodent models of impaired extinction have thus been generated with the objective of approximating this clinical, relapse prone aberrant extinction learning. These models have helped to reveal neurobiological correlates of extinction circuitry failure, gene variants, and other mechanisms underlying deficient fear extinction. In addition, they are increasingly serving as tools to investigate ways to therapeutically overcome poor extinction to support long-term retention of extinction memory and thus protection against various forms of fear relapse; modeled in the laboratory by measuring spontaneous recovery, reinstatement and renewal of fear. In the current article, we review models of impaired extinction built around (1) experimentally induced brain region and neural circuit disruptions (2) spontaneously-arising and laboratory-induced genetic modifications, or (3) exposure to environmental insults, including stress, drugs of abuse, and unhealthy diet. Collectively, these models have been instrumental in advancing in our understanding of extinction failure and underlying susceptibilities at the neural, genetic, molecular, and neurochemical levels; generating renewed interest in developing novel, targeted and effective therapeutic treatments for anxiety and trauma-related disorders.

*Canst thou not minister to a mind diseased,*

*Pluck from the memory a rooted sorrow,*

*Raze out the written troubles of the brain…*

Shakespeare, Macbeth

## Fear extinction: from Pavlov to the present

**Fig. 1:**
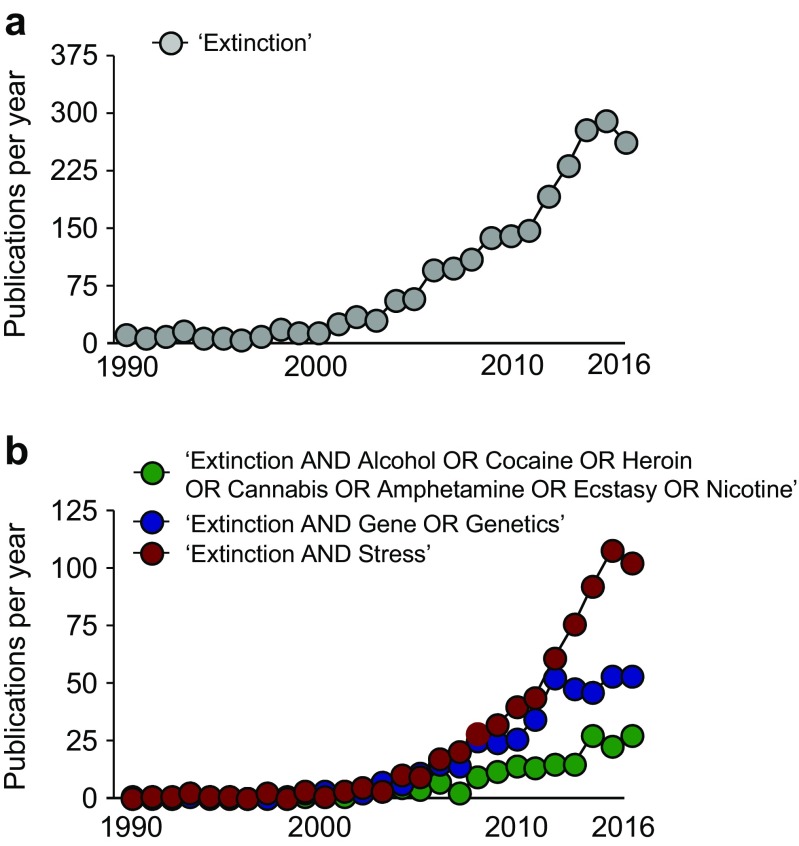
The growing popularity of rodent models of impaired fear extinction. A PubMed search was performed for the years 1990–2016 (inclusive), using the search term “Extinction” (**a**) or a combination of the terms “Extinction AND Alcohol OR Cocaine OR Heroin OR Cannabis OR Amphetamine OR Ecstasy OR Nicotine,” “Extinction AND Gene OR Genetics,” or “Extinction AND Stress” (**b**)

It is almost 100 years since I.P. Pavlov (1849–1936) described extinction as a phenomenon whereby repeated non-reinforced presentation of a conditioned stimulus (CS) led to the reduction in the magnitude of the conditioned response(s) (CR) (Pavlov 1927). In the case of *fear* extinction, the CS is typically a previously neutral stimulus that, through conditioning, has come to be associated with an aversive outcome, such that its occurrence alone is capable of eliciting some form(s) of fear/anxiety/defensive CR. The CR, and the degree of its diminution in amplitude and frequency under extinction, can be objectively measured and quantified in the laboratory in human (e.g., galvanic skin response, startle) and rodent (e.g., startle, freezing) subjects and has even been documented in invertebrates (Eisenhardt 2014). In part because of its conceptual appeal as an (ostensibly) straightforward psychological phenomenon and the relative ease of measurement across species in the laboratory, fear extinction has become an increasingly popular behavioral assay in clinical and preclinical settings alike (Flores et al. 2018; Hariri and Holmes 2015). Pavlov would no doubt be astonished at the number of research publications that now in some way make use of fear extinction (Fig. 1).

In addition to being relatively easy to compare readouts of fear extinction between humans and rodents, given the similarity of laboratory testing procedures, there are a number of other factors that give extinction translational appeal. First, as summarized below, many of the brain regions that have been connected in some way to effective or deficient fear extinction, respectively, are common across humans and rodents—lending credence to the idea that findings at the neural level in one species are informative to the other (Milad and Quirk 2012). Second, fear extinction has repeatedly been found to be impaired in various psychiatric conditions associated with trauma, notably PTSD, but also phobias, panic disorder, and obsessive-compulsive disorder (Lissek et al. 2005; Milad et al. 2008; Milad et al. 2009; Rauch et al. 2006; Rosen and Schulkin 1998; Wicking et al. 2016; Zuj et al. 2016). Indeed, poor fear extinction efficacy is linked to the persistence and treatment resistance of symptoms in PTSD (King et al. 2018b; Sijbrandij et al. 2013). Deficient fear extinction therefore represents a robust clinical endophenotype for these disorders and, as such, has particular significance in the current “age of RDoC” (Anderzhanova et al. 2017; Lonsdorf and Richter 2017). Third, extinction in rodents is impaired by known factors in the etiology of trauma-related conditions—we discuss some of these factors in greater detail below. Fourth, the extinction of unwanted responses to reminders of (a) prior trauma(s) is a core process underlying exposure therapy for PTSD and other anxiety disorders. Indeed, individual differences in fear extinction in humans are predictive of the degree of fear reductions produced by exposure therapy (Ball et al. 2017; Waters and Pine 2016) and enhanced extinction recall positively predicts cognitive behavioral therapy (CBT) outcome in, for example, social anxiety disorder (Ball et al. 2017; Berry et al. 2009; Forcadell et al. 2017).

## The importance of models of impaired extinction

**Fig. 2:**
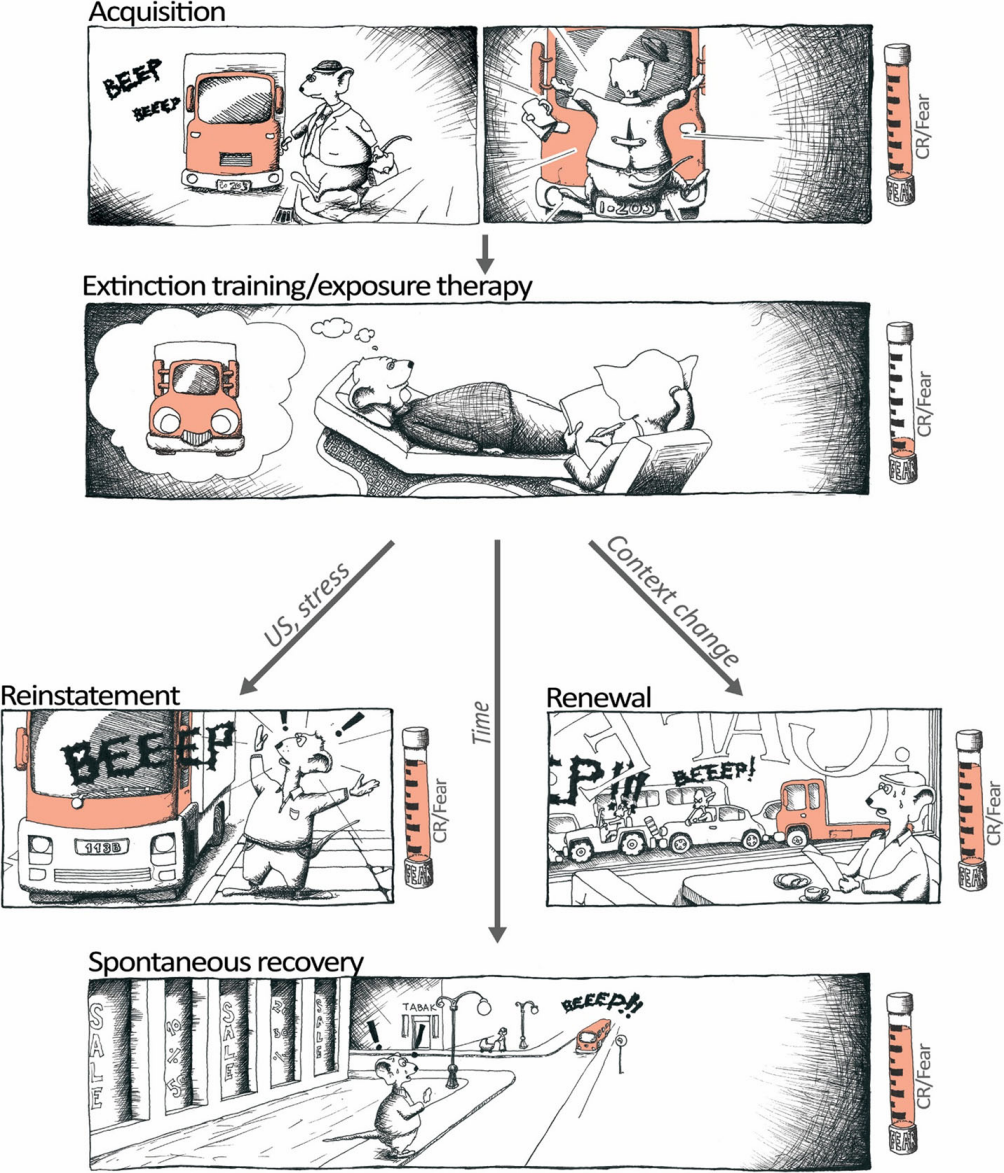
Relapse of extinguished fear poses an important challenge in behavioral, extinction-based therapies. This cartoon depicts three of the principal ways relapse can occur and which can be modeled in the laboratory both in animals and humans by return of fear paradigms. Reinstatement: the return of fear following exposure to the original US or stressors. Renewal: the return of fear following exposure to the original trauma-associated context or to contexts that otherwise differ from the therapy-context. Spontaneous recovery: the return of fear simply with the passage of time since therapy. CR conditioned response; US, unconditioned stimulus

The various facets of fear extinction support the face, construct and predictive validity of this measure as a behavior and higher-order neural process of relevance to both the pathophysiology and treatment of PTSD and other emotional disorders. There is a pressing need for such translationally relevant experimental paradigms because emotional disorders are at globally pandemic proportions (Craske et al. 2017) and resistance to current treatments remains a major constraint to recovery (Sippel et al. 2018). While exposure therapy can be successful in alleviating anxiety, fear extinction is an inherently fragile form of inhibitory memory that is prone to reinstatement (in the face of stressors), spontaneous recovery (with the passage of time) and renewal of fear (in non-extinction contexts) (Bouton 2014; King et al. 2018b; Vervliet and Raes 2013) (Fig. 2). These examples of fear relapse are observed in individuals having undergone exposure therapy and follow-up assessments. It has therefore been proposed that failure to build fear inhibitory associations can explain the high rates of fear relapse in anxiety disorder patients (Craske et al. 2014). The risk of relapse thus remains a major limitation of current therapies and advocates for the importance of models that capture not just the extinction process per se, but a scenario more closely approximating to the clinical, relapse-prone, clinical picture of impaired extinction.

Much has already been written on the topic of extinction. In the current article, our goal is not to attempt a summary of the vast literature pertaining to fear extinction. Rather, we aim to highlight some of the work that has been directed at studying and developing rodent models of impaired fear extinction. This is because as already noted, by recapitulating the deficient extinction present in anxiety and trauma-related disorders, these models arguably have the greatest potential to reveal insight into important aspects of the pathophysiology of these disorders. Furthermore, a better understanding of the mechanisms of impaired extinction and the associated limitations of current therapeutic strategies forms a solid platform for designing new approaches to more effective therapeutics (Bukalo et al. 2014; Graham et al. 2011; Singewald et al. 2015).

**Fig. 3:**
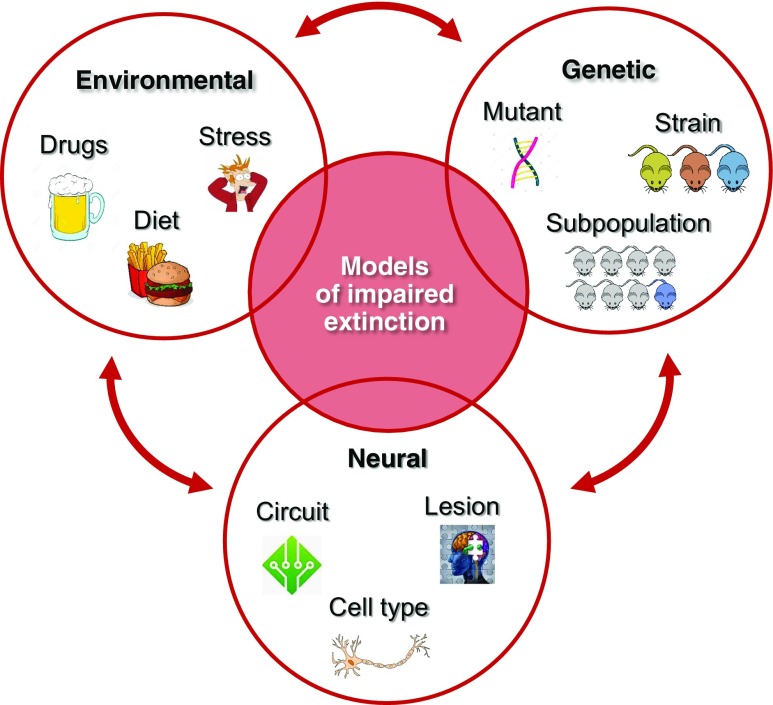
Some of the main classes of models of impaired extinction

We place models into somewhat arbitrary subcategories based on how the impairment in fear extinction was produced: by disruptions to neural function, via genetic engineering or spontaneous variations, or from other factors including environmental insults such as drug, bad diet, or stress exposure (Fig. 3). A “model” in this context is a subject exhibiting an impairment in extinction *as a result of* one (or more) of these factors, and is distinguished from the use of fear extinction as a test or assay (Cryan and Holmes 2005). We have not included models of impaired extinction of avoidance behavior and refer the reader to excellent recent review of this emerging literature (Rodriguez-Romaguera and Quirk 2017). We acknowledge from the outset that the scope of the article is far from exhaustive and does not cover a great deal of important research, particularly with regard to pharmacologically induced deficits in extinction already covered in earlier reviews (Giustino and Maren 2018; Singewald et al. 2015).

Nonetheless, relevant examples for each category are shown in Table 1, together with observed evidence of corresponding extinction-circuit abnormalities. In these examples and elsewhere, we dissociate impairments (i.e., delayed and/or no/insufficient reductions in conditioned responding) occurring either within extinction-training, from deficits in extinction memory retrieval manifest at recent (e.g., 1 day) or more remote (e.g., 1 week) timepoints after training. While the importance of within-session extinction for longer-term reduction in fear responding is debated (Plendl and Wotjak 2010), there is evidence to suggest that the rate of within-session extinction determines vulnerability to fear relapse (King et al. 2018b). In addition, at least some degree of within-session extinctions appears to be important for pharmacological boosting of extinction (Smits et al. 2013) (for further discussion, see Singewald et al. 2015).

**Table 1:** Representative examples of rodent models of impaired fear extinction. Effects on extinction training, retrieval (typically assessed 1 day after training) and fear relapse (assessed via spontaneous recovery, renewal, or reinstatement). Abbreviations: I = impaired, N = not impaired, n.d. = not determined, ↑ high freezing in fear relapse paradigm, - not changed. FC = fear conditioning, BDNF = brain-derived neurotrophic factor, BLA = basolateral nucleus of the amygdala, CeL = centrolateral nucleus of the amygdala, CeM = centromedial nucleus of the amygdala, dmPFC = dorsomedial prefrontal cortex, IFN-α = interferon alpha, LPS = lipopolysaccharide, SR = spontaneous recovery, vmPFC = ventromedial prefrontal cortex, 5-HTT = serotonin transporter

Model subcategory	Extinction training	Extinction retrieval	Fear relapse	Extinction circuitry dysfunction	Reference
Region and neural circuit disruptions					
BLA inactivation	(N)	I	n.d.		(Sierra-Mercado et al. 2011
vmPFC inactivation	I	I	n.d.		(Sierra-Mercado et al. 2011
vHPC inactivation	(N)	I	n.d.		(Sierra-Mercado et al. 2011)
CeL cell-specific disruption	N	I	n.d.		(Gafford et al. 2012)
Genetic factors					
5-HTT deletion	I	n.d.	n.d.	vmPFC/BLA dendritic dysmorphology, vmPFC hypoactivity	(Wellman et al. 2007)
BDNF mutation	I	n.d.	n.d.	vmPFC hypoactivity	(Soliman et al. 2010)
S1 inbred strain	I	I	↑	vmPFC/BLA hypoactivity, dmPFC/CeM hyperactivity	(Hefner et al. 2008; Sartori et al. 2016)
S1 (weak conditioning)	N	I			(Whittle et al. 2013)
Selection for trait anxiety	I	I	↑.	vmPFC hypoactivity, dmPFC hyperactivity	(Muigg et al. 2008; Yen et al. 2012)
Subpopulation stratification	I	I	↑	vmPFC/BLA dendritic dysmorphology	(Laricchiuta et al. 2016)
Exposure to environmental insults, developmental factors					
Acute stress	I	I	n.d.	vmPFC/BLA dendritic dysmorphology	(Maroun et al. 2013)
Single prolonged stress	N	I	↑	vmPFC hypoactivity, BLA/vHPC hyperactivity/connectivity	(Knox et al. 2012; Knox et al. 2018)
Acute stress and behavioral stratification	I	I	↑	vmPFC hypoactivity, BLA hyperactivity/gene expression	(Sillivan et al. 2017)
Subchronic or chronic stress	N	I	n.d.	vmPFC dendritic dysmorphology	(Izquierdo et al. 2006; Miracle et al. 2006)
Chronic ethanol	N	I	↑	vmPFC hypoactivity, dmPFC dendritic dysmorphology	(Holmes et al. 2012)
High fat/sugar diet	I(trend)	I	n.d.	Fewer vmPFC parvalbumin cells, increased vmPFC FosB/ΔFosB	(Baker and Reichelt 2016)
Immune activation (IFN-α)	I	n.d.	n.d.	BLA microglia, astrocyte activation	(Bi et al. 2016)
(LPS)	I	I	n.d.		(Quinones et al. 2016)
Adolescence	N	I	n.d.		(McCallum et al. 2010)
	I	I	-	vmPFC hypoactivity	(Hefner and Holmes 2007; Pattwell et al. 2013)

## Toward a neurotopography of extinction: system and circuit models of impaired extinction

Altering the activity of specific brain regions is a classical approach to mapping the key structures necessary for the acquisition, consolidation, or retrieval of extinction. Traditional methods such local lesioning, interregional disconnection, electrical stimulation, and pharmacological (e.g., GABA receptor agonist) inactivation, have been supplemented by newer optogenetic and chemogenetic approaches that allow for access over specific neural populations and pathways with better spatial and/or temporal precision. From these studies, it is clear that the learning of and the memory for extinction is distributed in a network fashion across brain areas including the prefrontal cortex (PFC), amygdala and hippocampus, but also a range of additional structures such as periaqueductal gray (PAG), bed nucleus of the stria terminalis (BNST), VTA, striatum, and others.

The broad strokes of the principal findings to date are as follows: Using a range of techniques, including temporary inactivations, immediate-early gene analyses, single-unit recordings, and optogenetics, activity in the dorsomedial PFC (dmPFC) positively associates with high fear/poor extinction, via reciprocal connections to pro-fear subpopulations of projections neurons in the basal amygdala (BA) (Burgos-Robles et al. 2009; Courtin et al. 2014; Dejean et al. 2016; Fitzgerald et al. 2014; Karalis et al. 2016; Senn et al. 2014; Whittle et al. 2010). Conversely, the ventromedial PFC (vmPFC) and its projections to the BA exerts a pro-extinction effect (Bloodgood et al. 2018; Bukalo et al. 2015; Sierra-Mercado et al. 2006). In turn, ventral hippocampus (vHPC) inputs to the central amygdala (CeA) (Xu et al. 2016) and vmPFC (Marek et al. 2018) are recruited to gate the flow of information underlying contextual fear and context-induced fear renewal after extinction, respectively.

The well-studied vHPC-mPFC-BA system subserving extinction is itself supported by other structures that are being revealed by recent work. These include the ventrolateral periaqueductal gray (vlPAG), which when inhibited impairs extinction (and fear learning) (Arico et al. 2017; Tovote et al. 2015), the BNST, inhibition of which prevents stress-induced fear renewal (Goode et al. 2015) and the lateral part of the central amygdala (CeL), wherein different populations of cells are predicted to promote or oppose the expression of extinction via inputs from the paraventricular nucleus of the thalamus (PVT) (Do-Monte et al. 2015; Haubensak et al. 2010; Knobloch et al. 2012; Li et al. 2013; Penzo et al. 2015). Thus, functional deficits at any of these multiple nodes within the highly integrated “extinction network” potentially contribute to extinction deficits (Knox et al. 2018) (reviewed in Holmes and Singewald 2013) and enhanced propensity for fear relapse (Marek et al. 2018) in psychiatric disorders due to irregularities in communication across the network. A recurring observation across a diverse set of models (Table 1) links functional deficiencies in certain nodes in the “extinction circuit;” in particular, emphasizing loss of function in the vmPFC (infralimbic cortex) and BLA and CeL subregions of the amygdala, and a corresponding over-engagement of the dmPFC (prelimbic cortex) CeM amygdala nucleus. For some examples of disruptions in fear extinction caused by experimental manipulations of these nodes (see Table 1**)**.

The notion of network disruptions underlying poor extinction is also already finding support from human functional imaging studies, though the low spatial resolution of these tools does not afford the same level of subregional changes revealed by the rodent studies (Fenster et al. 2018; Fullana et al. 2018; Sevenster et al. 2018). For instance, high resting dmPFC metabolism correlates with low vmPFC and hippocampal activation during extinction recall and this, in turn, associates with PTSD severity scores (Marin et al. 2016). Moreover, hippocampal-vmPFC co-activation in healthy subjects correlates with superior extinction recall (Kalisch et al. 2006; Milad et al. 2007; Rabinak et al. 2013), while stronger hippocampal–dmPFC connectivity is associated with greater fear renewal (Hermann et al. 2017). An important goal for future human and rodent studies is now to further parse precisely how these finely balanced dynamic interregional interactions breakdown during impaired extinction (Lesting et al. 2011; Muigg et al. 2008).

## It is (not) all in the genes: genetic models of impaired extinction

The fact that there is such high heritability estimates of PTSD and anxiety disorders (Pitman et al. 2012; Stein et al. 2002), suggests there is a genetic component to the risk of developing a clinical disorder after encountering trauma(s) (Almli et al. 2014). The most common (“reverse genetics”) approach to identifying genetic factors associated with impaired extinction has been to examine the behavioral consequences of engineering functional changes (e.g., knockout, knockin, overexpression) in specific genes encoding for molecules including Reelin, Pet-1, GAD67, Plaur, Dynorphin, GRP, Trk B, Stathmin, and others (Bukalo et al. 2014). Of those models based on well-known human polymorphisms, candidate genes associated with allele-specific variation in extinction (Lonsdorf and Kalisch 2011), include the BDNF Val66Met (see Felmingham et al. 2018 for evidence of a link between BDNF alleles and impaired fear extinction learning in PTSD) and COMT Val158Met polymorphisms (Table 1). Other examples are FKBP5 and the serotonin transporter, 5-HTTLPR, which interacts with stress to influence risk for PTSD (Caspi et al. 2010).

To date there have been fewer examples of models that are based on a ‘forward-genetics’ approach that use a rat or mouse strain exhibiting impaired extinction as a basis for elucidating underlying biological and genetic correlates (Holmes and Singewald 2013; McGuire et al. 2013). However, our laboratories have taken such an approach in examining a profound extinction deficit in a common inbred mouse strain, 129S1/SvImJ (S1), that was detected from a mouse inbred strain panel survey (Camp et al. 2009; Camp et al. 2012; Flores et al. 2014; Hefner et al. 2008; Temme et al. 2014). This inability to extinguish fear is seen across cued and contextual fear paradigms when contrasted with the profiles of normal extinguishing (e.g., C57BL/6 J) mouse strains. Interestingly, however, under “weak” (low shock) fear conditioning, short-term extinction acquisition is evident in S1 mice but the extinction memory still fails to consolidate and express over the long-term (Whittle et al. 2013).

The deficit in S1 mice does not extend to appetitively motivated instrumental extinction (Hefner et al. 2008), but there is deficient safety learning and overgeneralization of fear to ambiguous contexts and cues in these mice (Camp et al. 2012; Temme et al. 2014). Notably, deficient safety learning and fear overgeneralization are also characteristics of anxiety and trauma-related disorders (Duits et al. 2015; Lissek et al. 2014; Lissek et al. 2005). In another clinical parallel, S1 mice have lower heart rate variability (HRV) and depressed HRV during extinction training (Camp et al. 2012); resembling the reduced HRV seen in anxiety patients (Chalmers et al. 2014) and the slow recovery of HRV after trauma recall in PTSD patients (Arditi-Babchuk et al. 2009).

At the neural level, ex vivo immediate-early gene analysis (Hefner et al. 2008) and in vivo neuronal recordings (Fitzgerald et al. 2014) has revealed evidence of hyper-excitability in the dmPFC and medial nucleus of the CeA (CeM) and hypoactivity in the vmPFC and BA of S1 mice (Table 1), consistent with the respective pro-fear and pro-extinction roles of these regions. Speaking to the translational relevance of these observations, they align well with functional magnetic resonance imaging studies of patients with PTSD that reported a hypoactivation of the vmPFC and exaggerated amygdala reactivity during extinction recall (Garfinkel et al. 2014; Milad et al. 2009; Phelps et al. 2004). The generation of an effective extinction memory in these structures requires the expression and translation of relevant plasticity and learning-associated genes (Orsini and Maren 2012; Singewald et al. 2015).

The mechanisms by which the expression of genes are fine-tuned to, in turn, shape extinction is an emerging area that has also led to a focus on microRNAs (miRNAs) (Murphy and Singewald 2018)—a class of short, single-stranded non-coding RNAs (Smith and Kenny 2017). A pioneering study on this subject showed that extinction training increased the microRNA, miR-128b, to cause expression of a set of genes which are associated with synaptic plasticity. When miR-128b was experimentally increased in the IL of the B6 mouse strain, it was found to promote fear extinction (Lin et al. 2011). In an extinction-deficient mouse model, microarray approaches revealed that miR-144-3p exhibited increased amygdalar expression following successful extinction training. Viral enhancement of miR-144-3p expression in the BA rescued impaired fear extinction in S1 mice leading to reduced conditioned responses during both training and extinction retrieval. Furthermore, miR-144-3p overexpression protected against the return of fear in extinction-intact B6 mice, suggesting that miR-144-3p plays a critical role in extinction learning and long-lasting fear alleviation via interaction with its target genes *Pten*, *Notch1*, and *Spred1*, and their noted plasticity-associated downstream signaling cascades (Murphy et al. 2017).

Clearly, we remain in the earliest stages of defining how extinction efficacy is influenced not only by inherited gene variation, but also the ever-increasing range of mechanisms that are engaged to lay down extinction memories by controlling gene-expression. This is certainly an exciting area to watch going forward.

## Stress, drugs, and bad diet: environmental insult models of impaired extinction

We now turn to models of impairments in extinction produced by various environmental insults and certain other factors (Table 1). Given clinical evidence that a history of exposure to stress is a major risk factor for anxiety and trauma-related disorders, there have been multiple efforts to model stress-induced extinction deficits in rodents and identify mechanisms to prevent or reverse these (Chauveau et al. 2012) This literature has been recently reviewed (Deslauriers et al. 2018; Maren and Holmes 2015; Stockhorst and Antov 2015), but we would like to emphasize a number of the key findings here. One notable point is that the literatures on the neural and genetic correlates of impaired extinction increasingly align with emerging evidence linking stress and extinction. As an example, the aforementioned contrasting extinction phenotype of the S1 and B6 strains was exploited by a quantitative genetic approach to uncover a genomic region associated with extinction located on chromosome 3, and a novel candidate gene (peptidylprolyl isomerase D, *Ppid*) encoded within this genomic region (Gunduz-Cinar et al. 2018).

In turn, *Ppid* is a member of the tetratricopeptide repeat protein family, which includes FKBP5, and is involved in the regulation of steroid hormone receptors (Zannas and Binder 2014). Moreover, *Ppid* alters extinction in a manner requiring the glucocorticoid receptor (GR), suggesting this gene affects extinction by modulating a key stress-regulating system (Gunduz-Cinar et al. 2018). This latter finding is notable given an increasingly compelling translational evidence implicating glucocorticoids in trauma-related conditions and fear extinction (Maren and Holmes 2015) and S1 mice have abnormal HPA-axis responses to stress (Camp et al. 2012). For example, at least a subpopulation of PTSD patients show increased sensitivity of the negative-feedback system of the HPA-axis and lower cortisol levels (Yehuda 2002), while (systemic or intra-BLA) administration of GR agonists promotes extinction in rodents (Flores et al. 2018) and in humans with PTSD or other anxiety disorders (de Quervain et al. 2011; Michopoulos et al. 2017; Soravia et al. 2014).

Another major take home message in this section is that the effects of stress on extinction are dependent not only on the type and chronicity of the stressors, but also on the age, sex, and prior experience of the subject. For example, extinction efficacy varies dramatically across development and adolescence through into adulthood, both in rodents and in humans (Baker et al. 2016; Pattwell et al. 2012) (Table 1). Concerning stressor-type, to date, it seems that immobilization stress and single prolonged stress produce particularly robust deficits in fear extinction (Deslauriers et al. 2018). In an illustrative example of the often reported nuances in this field, stressing adolescent rats, through a combination of predator order and elevated platform exposure, impaired extinction into adulthood but, for reasons that remain unclear, did so only in males and not females (Ter Horst et al. 2012; Toledo-Rodriguez et al. 2012). This extends clear evidence of sex differences in fear extinction (Baran et al. 2009; Fenton et al. 2016; Hunter 2018; Matsuda et al. 2015; Shansky 2015; Shvil et al. 2014).

Adding to the complexity of this area, not only are there varying responses to stress between sexes, but there is also individual variation within a population of the same sex. For example, following exposure to a footshock-based stress-enhanced fear learning procedure, male (but not female) (C57BL/6 J) mice could be split into extinction-resilient and susceptible subgroups, that were in turn associated with specific patterns of corticoamygdala activity (Table 1) and gene expression (Sillivan et al. 2017). These data advocate for the greater consideration of subpopulation differences in stress-related models of impaired extinction, both to better approximate the marked individual differences in risk for stress disorders in humans and predict the efficacy of drugs and other therapeutic interventions (King et al. 2018a).

There are interesting overlaps between the effects of exposure to stress and drugs of abuse, another risk factor for anxiety disorders and PTSD, on extinction. For example, chronic cannabis use is associated with impaired extinction in humans (Papini et al. 2017) as is chronic ethanol exposure in rodents. Socially isolating rats during adolescence has been shown to increase ethanol drinking and impair fear extinction (Skelly et al. 2015), while chronic exposure to vaporized ethanol impairs extinction retrieval in mice, in association with dendritic dysmorphology and blunted NMDA receptor-mediated neuronal transmission in the dmPFC (Holmes et al. 2012). Along similar lines, 2 weeks of ethanol consumption in a liquid diet rendered rats extinction resistant (Bertotto et al. 2006), while a shorter regimen of intraperitoneally administered ethanol produced an increase in fear during extinction acquisition and increased neuronal activation (i.e., c-Fos expression) in the dmPFC, BA, CeA, and PVT (Quinones-Laracuente et al. 2015). However, while stress and ethanol exposure may produce similar effects on extinction, there is no clear cut relationship between differences in the propensity to drink ethanol across mouse strains and the capacity for extinction (Crabbe et al. 2016).

Unlike most other abused drugs, alcohol is consumed like a food and is a source of calorific intake. The degree to which this contribute to the deleterious physiological effects of chronic drinking is debated but may be relevant here given recent evidence that abnormal diet can affect fear extinction. Rats fed a high-fat/high-sugar diet over the course of 6 weeks developed poor extinction (Table 1) and altered certain makers of infralimbic cortex function (Baker and Reichelt 2016). One possibility is that such effects are driven by the neuroinflammatory responses that are associated with a high-fat diet (Valdearcos et al. 2014). Giving credence to this hypothesis are reports that immune activation (via lipopolysaccharide administration) disrupts fear extinction (Quinones et al. 2016), as does intra-BLA infusion of interferon-α; in a manner preventable by administration of a microglial activation inhibitor (minocycline) (Bi et al. 2016).

We wish to highlight these latter findings not to overstate the potential importance of poor diet as a risk factor for trauma-related conditions, but rather to underscore the expanding range of environmental insults found to disrupt fear extinction. A challenge for future work will be to model the real-world combination of dietary factors, exposure to drugs and life stressors faced by most at-risk individuals, and to use the rodent models to decipher how these interact and potentially synergize to affect clinical outcomes.

## Outlook: using models of impaired extinction to discover novel therapeutic strategies

The value of rodent models of impaired extinction is ultimately gauged by their utility as a platform for the identification of novel mechanisms for therapeutically normalizing extinction. While there are examples of targeting circuit abnormalities via deep brain stimulation in these models (Rodriguez-Romaguera and Quirk 2017; Whittle et al. 2013), much of this work has focused on pharmacological approaches, reflecting the continued importance of developing novel drugs for anxiety, and trauma-related disorders (Bukalo et al. 2014; Graham and Richardson 2011; Singewald et al. 2015). Encouragingly, certain models in mouse strains (e.g., S1 strain (Gunduz-Cinar et al. 2013; Gunduz et al. 2015; Hefner et al. 2008; Sartori et al. 2016; Whittle et al. 2010; Whittle et al. 2016; Whittle et al. 2013) and rats, e.g., ethanol-exposed (Bertotto et al. 2006), stressed (Matsumoto et al. 2013), and adolescents (Ganella et al. 2017; McCallum et al. 2010), have demonstrated that deficient extinction is effectively rescued by pharmacological manipulations of various transmitter systems including serotonergic, glutamatergic, dopaminergic, noradrenergic, endocannabinoid signaling. Along these lines, there is the exciting potential for enhancing disturbed neuroplasticity in extinction-related circuits via epigenetic mechanisms such as histone acetylation (Whittle et al. 2016; Whittle et al. 2013) or altering the expression of specific microRNAs, such as miR144 (Murphy et al. 2017) to produce long-term fear reductions in extinction-impaired subjects.

Once a promising pharmacological target is identified, a critical question is how it should be clinically administered to maximize its therapeutic potential and mitigate risk of failure in clinical trials. In this regard, the administration of a single drug concomitant to fear extinction in extinction-deficient individuals does often not suffice to support the extinction memory-augmenting mechanism to an extent that prevents temporal, spatial, or stress-dependent fear relapse (Singewald et al. 2015). Using extinction-deficient mice, our group was able to show for the first time that only the administration of neuropeptide S (NPS) before and the NMDA receptor partial agonist d-cycloserine (DCS) after successful extinction training, but not administration of NPS alone results in formation of a robust extinction memory, which withstands various types of fear relapse (Sartori et al. 2016).

Supporting the utility of this dual pharmacotherapeutic concept, it was demonstrated that fear relapses in extinction-deficient mice can also be reduced by combined administration of L-DOPA and the HDAC-inhibitor MS-275, concomitant to extinction training (Whittle et al. 2016). This speaks to the potential importance of dual or multiple pharmacotherapeutic adjuncts to extinction in these cases. These should be critical considerations in drug development when designing preclinical experiments to evaluate translational potential in extinction-impaired rodent models. Moreover, at present no drug that can pass the blood brain barrier has a pharmacodynamic profile that combines the advantages of promoting memory and reducing anxiety, without also being sedating. This is not to say it is unfeasible and has already been achieved by neuropeptide S or fibroblast growth factor-2 targeting drugs, (Graham and Richardson 2011; Sartori et al. 2016) and endocannabinoid-targeting approaches (Micale et al. 2013; Patel et al. 2017), to give just a few examples. Although the pharmacological augmentation of exposure-based therapies has not yet entered broad clinical use, it represents an exciting idea with the clear potential for improving clinical outcome.

Beyond pharmacological approaches, less conventional approaches have to date been less studied in extinction-deficient models, but are certainly worthy of investigation. One interesting modification to changing the way extinction memories are formed simply involves training in multiple contexts (de Jong et al. 2018). This could potentially mitigate against the context dependency of extinction memories (Bukalo and Holmes 2018; Maren et al. 2013) and the high rate of fear relapse after CBT (Boschen 2009). Preliminary clinical work has shown that performing exposure therapy in multiple contexts reduces, for instance, the reoccurrence of fear of spiders (Vansteenwegen et al. 2007). As more extinction trials/longer CSs are typically needed to achieve reductions in fear in extinction-impaired individuals, behavioral manipulations that could potentially shorten these procedures are of particular interest. Reactivation of the original fear memory prior to or during extinction training has been proposed to render fear memories plastic and receptive to extinction (Monfils et al. 2009; Schiller et al. 2010) (but see Luyten and Beckers 2017) and has been successfully used to attenuate remote fear memories, which are known to be resilient against extinction-mediated attenuation (Khalaf et al. 2018). Along similar lines, there are neurally based strategies for reversing plastic changes underlying fear memory to enable extinction, including the targeting of perineuronal nets around parvalbumin-positive interneurons in the BA (Gogolla et al. 2009; Gunduz-Cinar et al. 2018).

A final point to underscore is that eventual success of novel treatments will be bolstered by grounding them in a solid understanding of how they act at the neural level. The field can draw upon the great advances that have been made in delineating the neural circuitry of fear extinction, as discussed above (Hariri and Holmes 2015). In the ideal scenario, extinction rescuing effects in an impaired model can be aligned with the normalization of disturbed neurobiological markers, including abnormal patterns of brain activation within key brain substrates for extinction (for an example, see Whittle et al. 2010). This notion of therapeutic circuit modulation is supported by clinical observations that successful exposure-based CBT is associated with the reversal of dACC and amygdala hyper-reactivity (Ball et al. 2017; Goossens et al. 2007; Straube et al. 2006) and improved extinction recall is associated with increased vmPFC activity (Ball et al. 2017; Lonsdorf et al. 2014; Milad et al. 2007). Though still preliminary, these convergent neural and behavioral data, from both the laboratory and clinic, help position models of impaired fear extinction as a vital component of future research aimed at developing effective new therapeutic approaches to alleviating the suffering of patients with trauma-related conditions.
